# Regional analysis of UK primary care prescribing and adult service referrals for young people with attention-deficit hyperactivity disorder

**DOI:** 10.1192/bjo.2019.94

**Published:** 2020-01-06

**Authors:** Anna Price, Tamsin Ford, Astrid Janssens, Andrew James Williams, Tamsin Newlove-Delgado

**Affiliations:** Research Fellow, College of Medicine and Health, University of Exeter, UK; Professor of Child and Adolescent Psychiatry, University of Cambridge, UK; Associate Professor, Department of Public Health, University of Southern Denmark, Denmark; and Honorary Associate Professor, University of Exeter Medical School, UK; Lecturer, European Centre for Environment and Human Health, University of Exeter, Knowledge Spa, Royal Cornwall Hospital, UK; Senior Clinical Lecturer and Honorary Consultant in Public Health, College of Medicine and Health, University of Exeter, UK

**Keywords:** CPRD, ADHD, transition, prescribing

## Abstract

**Background:**

Approximately 20% of children with attention-deficit hyperactivity disorder (ADHD) experience clinical levels of impairment into adulthood. In the UK, there is a sharp reduction in ADHD drug prescribing over the period of transition from child to adult services, which is higher than expected given estimates of ADHD persistence, and may be linked to difficulties in accessing adult services. Little is currently known about geographical variations in prescribing and how this may relate to service access.

**Aims:**

To analyse geographic variations in primary care prescribing of ADHD medications over the transition period (age 16–19 years) and adult mental health service (AMHS) referrals, and illustrate their relationship with UK adult ADHD service locations.

**Method:**

Using a Clinical Practice Research Datalink cohort of people with an ADHD diagnosis aged 10–20 in 2005 (study period 2005–2013; *n* = 9390, 99% diagnosed <18 years), regional data on ADHD prescribing over the transition period and AMHS referrals, were mapped against adult ADHD services identified in a linked mapping study.

**Results:**

Differences were found by region in the mean age at cessation of ADHD prescribing, range 15.8–17.4 years (*P*<0.001), as well as in referral rates to AMHSs, range 4–21% (*P*<0.001). There was no obvious relationship between service provision and prescribing variation.

**Conclusions:**

Clear regional differences were found in primary care prescribing over the transition period and in referrals to AMHSs. Taken together with service mapping, this suggests inequitable provision and is important information for those who commission and deliver services for adults with ADHD.

## Background

Rates of primary care prescribing of attention-deficit hyperactivity disorder (ADHD) medication for young people with ADHD in the UK appear to decline more steeply than expected given the rate of symptom reduction from follow-up studies.^1^ The timing coincides with the age at which children's services end, usually between 16 and 18, and transition into adult services occurs if continuation of medication is recommended. This is a key developmental stage when multiple other transitions are likely, such as changing educational setting or leaving home for the first time. The National Institute for Health and Care Excellence (NICE) guidelines recommend that prescribing of ADHD medication for adults should happen via shared-care agreements between primary and secondary care.^2^ The principle of shared care assumes that the young person will be in the care of an adult mental health service (AMHS) with the general practitioner (GP) continuing to prescribe.^3^ However, problems could arise, because of the lack of adult ADHD services, because there is no shared-care agreement or because GPs are not prepared, trained or supported to prescribe.^4,5^

Evidence is increasingly emerging of low rates of successful transition and poor-quality transition experiences for people with ADHD.^6,7^ Disruption of care during transition adversely affects young people with mental health conditions^8^ whereas untreated ADHD can worsen health, education and occupational outcomes.^9,10^ For those young people who continue to require medication, cessation of prescribing could be related to lack of service provision. Studies have suggested that there are gaps in provision of adult ADHD services and a shortage of specialist services.^11,12^ Population prescribing studies suggest higher incidences of ADHD diagnosis and prescribing in young people in areas of socioeconomic deprivation,^13^ however, this is unsurprising given the strong link between deprivation and the prevalence of ADHD.^14^

## Aims

To our knowledge, no study has examined regional differences in prescribing for young people with ADHD in the UK during the transition period. Knowledge and understanding of any regional variations in prescribing rates through the transition, and potential links to availability of dedicated adult ADHD services may allow commissioners and practitioners to address inequalities of provision. The current study aimed to analyse regional variation in prescribing patterns of ADHD medication and rates of referrals into AMHSs for young people with ADHD aged between 16 and 20 from 2005 to 2013, using the Clinical Practice Research Datalink (CPRD). It also aims to explore relationships between prescribing patterns, referral rates and service locations.

## Method

### Data source

The CPRD is a large database of anonymised patient records including diagnoses, prescribed drugs and referrals to secondary care services. The primary care section is contributed to by over 670 GP practices across the UK and contains the records of over 11 million patients. It covers up to 6% of the UK population and is broadly representative in terms of age, gender, ethnicity and geographical location of practices.^15^

The data source for service locations was the 2018 UK mapping study, a national survey of adult ADHD services, run as part of the ‘Children and Adolescents with Attention Deficit Hyperactivity Disorder (ADHD) in Transition from Child to Adult services’ (CATCh-uS) study of transition in ADHD.^16^ Data on services were gathered from over 2600 informants from across the UK; patients, health workers and National Health Service (NHS) commissioners. Data were collected via an online survey, freedom of information requests to commissioners (90% response rate) and surveillance reports. A total of 44 NHS dedicated adult ADHD services were identified, consisting of 29 ADHD; 7 ADHD and autism spectrum disorder; and 8 neurodevelopmental services.^17^

### Study design and population

We used a cohort from the CPRD of young people aged between 10 and 20 in 2005, for the study period that ran from 1 Jan 2005 until 31 Dec 2013.^1^ This allowed us to study prescribing over the transition period (see Statistical analysis below). To be included, individuals had to have a diagnosis of ADHD coded in their primary care record (*n* = 9390). Of included patients, 98.6% (*n* = 9261) had their first ADHD clinical code coded under the age of 18. ADHD diagnoses were defined as any of the 22 CPRD medical codes and primary care Read terms (based on ICD-10 F90 or DSM categories) that relate to an ADHD diagnosis (see Newlove-Delgado *et al*^1^ for details on patient identification and supplementary material). Patients were defined as having an ADHD prescription if any prescription record had an ADHD-related medication code, including categories of stimulants and non-stimulants for ADHD.^1^ Rare cases of patients with narcolepsy, for which ADHD medication may be prescribed (*n*<20) were excluded from the analysis.

Regions were defined by the NHS strategic health authority (SHA) boundaries, in which reporting GP practices were situated during the study period.

### Statistical analysis

The first phase of analysis used Stata version 15.0^18^ and focused on changes in prescribing of ADHD medication through the transition period (defined as 16–19 years of age) and incidences of referral to AMHSs. The CPRD did not supply dates of birth, therefore age bands were assigned, with, for example, age-band 14/15 indicating the year of their 15th birthday. We defined this transition period as being between the year of their 16th birthday, which commonly marks the end of children's services, and the year of their 19th birthday, which is when the transition to adult services should be completed, according to NICE guidance.^19^

First, analyses were carried out to examine differences in prescribing prevalence by region. The proportion of patients with an ADHD prescription at any point was calculated, followed by the proportion of others with a prescription in each age band from 14/15 years to 19/20 years, to cover the transition period and 1 year either side. For each region, the difference in the proportion of patients with an ADHD prescription between the beginning and end of the transition period was then reported. The denominator for each age band only included patients who had records for the full year in question.

Age at cessation of medication by region was then examined, with cessation defined as a gap of more than 6 months in prescriptions. This was chosen to allow for uncertainty in estimating prescription length as ADHD prescriptions are typically provided for between a 1- and 2-month duration, and to account for any medication ‘breaks’ that may occur. When calculating age at cessation, the cases of patients that were censored, as they still had a prescription at the time of leaving the database (i.e. lost to follow-up or at the age boundary of the cohort) were excluded from the analysis. As full details of date of birth are not provided by CPRD, to calculate the age of cessation date of birth was designated as 1 July for each individual, minimising error each way to a maximum of 6 months. Mean age of cessation was calculated with confidence intervals, and a one-way ANOVA run to explore differences in means by region.

Patients were defined as having a referral to an AMHS if they were coded with a referral to adult psychiatry, a community psychiatric nurse or clinical psychology. The proportion of patients referred by region was calculated, examining differences in proportions using the χ^2^-test. Given that referral might have been for non-ADHD related treatment, we also described the proportion excluding the cases of patients potentially referred for a psychiatric comorbidity. The first definition used was ‘cases without any other psychiatric diagnoses’. However, as diagnoses are not coded as reliably as prescription data in the CPRD,^20^ and common comorbidities such as autism spectrum disorder are not consistently treated in AMHSs, a second definition was also used of ‘cases without a prescription for any other psychotropic medication’.

Linear regression was used to examine the association between region (independent variable) and the age of cessation (dependent variable) and subsequently adjusted for referral to an AMHS as a covariate. The second phase of analysis used a geographic information system, QGIS 2.18, to analyse and display the service mapping data alongside the prescribing data. Shapefiles for UK countries and SHA regions were imported^21^ and maps created to illustrate changes in patterns of prescribing and rates of referral to AMHSs by region. Locations of dedicated NHS services for adults, as identified in the 2018 CATCh-uS study^17^ were plotted on the same maps.

We assert that all procedures contributing to this work comply with the ethical standards of the relevant national and institutional committees on human experimentation and with the Helsinki Declaration of 1975, as revised in 2008. All procedures involving human patients were approved by the following: for the CPRD data-set, the Independent Scientific Advisory Committee on behalf of the National Research Ethics Service Committee (protocol number 13_213); for the 2018 CATCh-uS mapping study, the University of Exeter Medical School Ethics Committee (REC Application Number: 15/07/070).

## Results

There were 9390 eligible patients: 84% (*n* = 7876) were male, 25% (*n* = 2335) had a recorded diagnosis of a psychiatric comorbidity, 62% (*n* = 5780) had at least one recorded prescription for an ADHD medication, and 25% (*n* = 2336) had a prescription for any other (non-ADHD) psychotropic medication. Following censoring, 3476 patients (37%) had complete data that allowed us to calculate age of cessation of ADHD medication. Patients who were censored were similar to those that were uncensored with respect to medication duration and year of birth.

### Percentage of patients with ADHD with an ADHD prescription

**Fig. 1:**
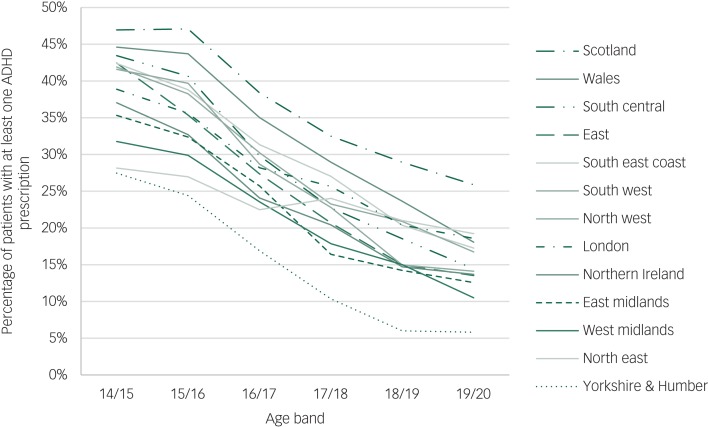
Percentage of patients with attention-deficit hyperactivity disorder (ADHD) with an ADHD prescription, by age band and region.

Scotland was the region with the highest percentage of patients (75%) with at least one ADHD prescription at any point, whereas Yorkshire and the Humber had the lowest (48%). There were differences by region in the proportion of patients that had an ADHD medication prescribed for every age band, see Fig. 1.

### Difference in proportion of patients with an ADHD prescription before and after transition

The drop in the proportion of patients with an ADHD prescription between the year of their 16th birthday and the year of their 19th birthday (age bands 15/16 and 18/19) was 19% for all patients; but varied by region from 6% in the North East to 25% in the North West, see Table 1 and Fig. 1.

**Table 1:** Difference in attention-deficit hyperactivity disorder (ADHD) prescriptions, mean age of cessation of ADHD medication, and instances of referral to adult mental health services (AMHSs); by subgroup and region

Region	Difference in % with ADHD prescription pre- and post- transition (15/16–18/19 years)	Age of prescription cessation, mean (95% CIs)	Percentage referred to an AMHSs (%)		
			All patients	Patients without any other psychiatric diagnosis	Patients without prescription for any other psychotropic medication
North East	6	17.4 (16.5–18.2)	5	3	3
North West	25	16.6 (16.4–16.9)	11	10	8
Yorkshire & Humber	18	15.8 (15.4–16.3)	8	8	6
East Midlands	18	16.5 (16.1–17.0)	4	3	4
West Midlands	15	16.1 (15.7–16.4)	11	10	8
East of England	20	16.2 (15.9–16.4)	8	7	6
South West	17	16.9 (16.6–17.2)	7	7	6
South Central	22	16.6 (16.3–16.8)	11	9	6
London	15	17.4 (17.0–17.8)	11	8	6
South East Coast	18	16.6 (16.3–16.8)	13	11	9
Northern Ireland	18	15.9 (15.4–16.4)	13	10	8
Scotland	18	16.9 (16.7–17.2)	10	8	7
Wales	20	16.6 (16.3–16.9)	21	15	11
Total	19	16.6 (16.5–16.7)	11	9	7

### Mean age of cessation of ADHD medication prescription

The mean age of termination (or interruption) of medication prescription was 16.6 years (s.d. = 2.63, 95% CI 16.5–16.7) (Table 1). A one-way ANOVA determined that differences in the mean age of end of ADHD medication prescription were statistically significant by region, with mean ages ranging from 15.8 to 17.4 years, *F*(12, 3463) = 6.18, *P*<0.001.

### Proportion of patients referred to AMHSs

The percentage of patients with ADHD referred to any AMHS was 11% (Table 1), but varied by region from 4 to 21%, χ^2^(12, *n* = 9390) = 121.60, *P*<0.001. When patients with any other non-ADHD psychiatric diagnosis were excluded from the data-set, the overall proportion dropped to 9%, and varied by region from 3 to 15% (χ^2^(12, *n* = 7055) = 49.12, *P*<0.001). When patients with a prescription for any other psychotropic medication were excluded, the percentage of those referred was even lower (7%), and also varied by region from 3 to 11% (χ^2^(12, *n* = 7052) = 27.73, *P*>0.001).

There was a marginal association between region and the age of cessation of ADHD medication prescription in the unadjusted model (*R*^2^ = 0.0013, *F*(1, 3474) = 4.56, *P* = 0.03). However, when referral to any AMHS was added to the model, (*R*^2^ = 0.0046, *F*(2, 3473) = 83.55, *P*<001), region was no longer a predictor and only referral to any AMHS was a significant predictor of age of prescription cessation.

### Regional variations, mapped against service locations

**Fig. 2:**
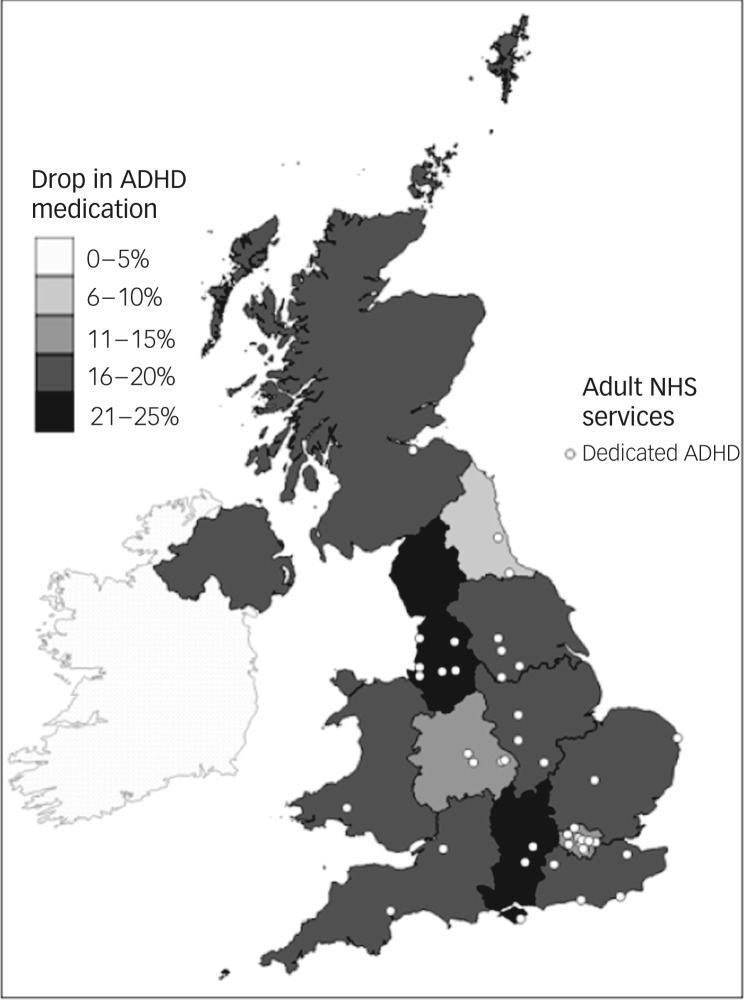
Drop in prescribing rates for attention-deficit hyperactivity disorder (ADHD) medication for young people with ADHD, between the age bands of 15/16 and 18/19: plotted against locations of dedicated adult ADHD services.

**Fig. 3:**
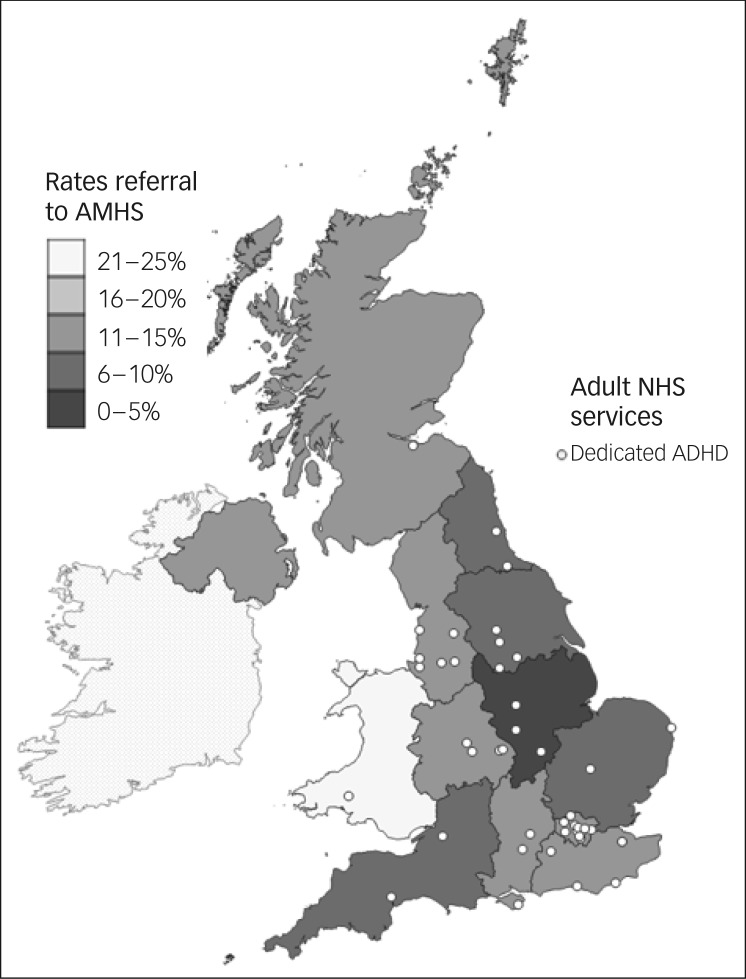
Referral rates to adult mental health services (AMHSs) for young people with attention-deficit hyperactivity disorder (ADHD), plotted against identified locations of dedicated adult ADHD services.

Figures 2 and 3 clearly illustrate the regional variations in prescribing patterns, referral rates and service locations. On visual inspection, however, there were no clearly identifiable relationships between reductions in prescribing, referral rates or identified locations of dedicated adult ADHD services.

NHS, National Health Service.

NHS, National Health Service.

## Discussion

We detected regional variations in primary care prescribing of ADHD medication for young people with ADHD through the transition period, and in the proportions of young people with ADHD being referred to an AMHS. The creation of visual maps showed clear variation by region for prescribing, referrals and the location of dedicated services, but no discernible relationships between these three measures. Distribution of services was patchy and uneven across the UK and there were many areas without a dedicated service nearby. These findings are novel, providing evidence of potential inequalities in healthcare provision across the UK for this patient group, which are likely to have an impact on continuation of care and treatment into adulthood.

### Organisation and availability of adult ADHD services

All young people included in this study had a diagnosis of ADHD in their primary care record; therefore, the findings of significant variation are unlikely to be because of regionally patterned differences in prevalence. Variations in prescribing and in referrals found by region are therefore likely to be related to differences in the organisation and availability of services for ADHD. There is currently no established or consistent approach to the configuration of health services for adult ADHD. Management in secondary care may be undertaken in generic AMHSs or within a specialist service,^22^ consequently variations found in primary care prescribing through transition may reflect a mixture of service models. Whether or not a young person has psychiatric comorbidities can also affect whether they are eligible for AMHSs, or directed to specialist or generic services.^23^ This may contribute to our findings of differences in referral rates for individuals without a comorbidity, or other psychotropic medication.

The service mapping we present also demonstrates a patchy geographical spread of specialist adult ADHD services. NICE recommends diagnosis and treatment for adults via separate teams or clinics with expertise in ADHD, but with no guidance on the size and time commitment.^2^ The NHS is structured differently in the four countries that comprise the UK, including different commissioning arrangements.^24^ There is an argument that treatment within generic services, which are not identified on the map, is potentially a cost-effective and practical solution to long-term treatment of ADHD, however, issues need to be addressed, such as a lack of training of AMHS professionals and the fact that services are often set up to provide episodic care, rather than to treat long-term conditions.^23^

### Variation in primary care practice

Our findings on variation in prescribing may also reflect regional differences in primary care practice and culture to some extent, however, we were unable to examine variation by clinical commissioning group (see Limitations). Although medication has been recommended by NICE for management of adult ADHD, as the 2008 guidance evidence suggests GPs may feel unsupported to prescribe ADHD medications to adults, with issues such as lack of training or lack of available support from a specialist service identified as barriers to prescribing.^4^ For example, a study in Northern Ireland reported reluctance from GPs to prescribe under a shared-care partnership, which may bear some relation to our finding that Northern Ireland had the earliest mean age of medication cessation in our analysis.^25^

### Transitions into adult ADHD services

Regardless of local prescribing and secondary care arrangements, if a young person does not transition into adult services, they are unlikely to continue to receive treatment into adulthood. Young people with ADHD are already at a higher risk of a range of negative health, educational and occupational outcomes compared with the general population, and without treatment, these risks are increased.^9,10^ The regional differences in prescribing and referrals found in this study support available qualitative evidence of unsupported transitions and findings of limited adherence to and inconsistencies in implementation of the NICE guidelines on transition.^7,11,12^

### Strengths and limitations

Strengths of this study include a large population-based sample of primary care records. To our knowledge this is the first time primary care ADHD medication prescribing in the transition age group among adults with a childhood diagnosis and referrals to AMHSs have been analysed by region. It is also the first time regional quantitative data on primary care prescribing and referral rates have been explored in comparison with national UK data on the locations of adult ADHD services. A key limitation of this was the time lapse between the study period for CPRD data (2005–2013) and the date of the service mapping study (2018). The provision and organisation of mental health services are constantly evolving and some services identified in 2018 may only have been recently commissioned. In addition, although the CPRD data-set automatically includes all primary care prescriptions, those issued through a secondary mental health service may be missing.^15^

Although we were able to analyse patterns of regional variation, we had no information on the severity of the ADHD diagnosis for each patient, and could not determine whether prescribing decisions were clinically appropriate or inappropriate. We were also unable to adjust for continuing symptom severity over time. There is considerable evidence emerging from qualitative and quantitative research of the premature cessation of ADHD medication through transition,^1,26,27^ which taken with evidence of regional variations in prescribing, are indicative of gaps in provision. But it is also possible that evidence of higher rates of continued prescribing at age 17/18 in some regions might reflect the cases of patients where a failure to review need had led to an inappropriate continuation of prescribing.

NICE^2^ guidance states that once a young person is stable on ADHD medication, prescribing should be through shared care, however, in some patients with complex cases secondary services may have prescribed for longer than the 6-month time period defined as medication cessation in this study. If this prescribing is not recorded in CPRD these patients may have been inaccurately recorded as having stopped medication. In addition, we may have underestimated referral rates, as although CPRD records referrals from primary care into AMHSs, if referrals are made using free-text letters these can only be captured by scanning the free text, which we did not have access. Similarly, free-text references to referrals between child services and AMHSs would not be included. However, this limitation is likely to apply across all regions, and is unlikely to have influenced the significant variation in referral rates found in our analysis.

A further study weakness is the size of regions analysed. Although SHA reflected the structure by which healthcare was organised during the CPRD study time period, their large size means they include multiple NHS trusts and many GP practices, which may vary in their arrangements for adults with ADHD. The size of defined regions also made it difficult to incorporate geographic deprivation measures into the analysis, as deprivation varies at much smaller geographies than this. Future research would benefit from using smaller geographic areas.

Finally, our sample frame was young adults with a childhood diagnosis of ADHD. Another important gap in adult services for ADHD is assessment, diagnosis and treatment for those who present as adults for the first time. Given our sample frame, we cannot comment on this important aspect of access to services, which should be addressed using an alternative sample frame. It would be interesting to see if regional variation in this related group of patients followed a similar regional pattern.

### Implications

In conclusion, these findings, combined with evidence that more than one in ten commissioners are failing to meet expected Mental Health Investment Standards, point to unequal provision of resources for mental health.^28^ Large and unchanging regional health inequalities in England point to the need for targeted interventions to improve the equity of access to care more generally.^29^ Studies of variation can help to increase accountability for mental health service provision, and indexing regional differences in prescribing and referral rates is one way of highlighting inequity. This data can contribute to planning regional service development and provision and, ultimately, to addressing health inequalities in people with ADHD.

## Data Availability

Data is currently stored securely by the University of Exeter Medical School, under embargo until the end of the CATCh-uS project.
